# Preliminary Impacts of a 3-Month School-Based Exercise and Data Recording Intervention on Lifestyle Habits, Psychological Health, and Physical Health in Japanese Adolescents

**DOI:** 10.3390/children13080980

**Published:** 2026-07-23

**Authors:** Yasuaki Kusumoto, Eri Takahashi, Yasuhiro Endo, Kanako Okazaki, Akihiko Asao, Yoshinobu Tanaka

**Affiliations:** 1 Department of Physical Therapy, School of Health Sciences, Fukushima Medical University, Fukushima City 960-8516, Japan; eritkhs@fmu.ac.jp (E.T.); ysendo@fmu.ac.jp (Y.E.); kana-oka@fmu.ac.jp (K.O.); 2 Department of Occupational Therapy, School of Health Sciences, Fukushima Medical University, Fukushima City 960-8516, Japan; a-asao@fmu.ac.jp (A.A.); tanaka-y@fmu.ac.jp (Y.T.)

**Keywords:** exercise, health support, screen time, self-efficacy, students

## Abstract

**Highlights:**

**What are the main findings?**

• A 3-month school-based intervention combining short exercise sessions and daily digital recording was associated with a decrease in daily screen time among Japanese adolescents who initially spent 4 or more hours per day on screens.
• The brief regular physical activity program was accompanied by an increase in the participants’ body mass index (BMI) and trunk muscle mass during the winter season, although the single-arm design limits definitive causal attribution.

**What are the implications of the main findings?**

• Integrating brief exercise with digital self-monitoring via school-provided iPads represents a feasible framework for helping students manage excessive screen time and maintain muscle mass within regular school schedules.
• Implementing even short-duration physical exercises within regular school hours may help counter wintertime physical deconditioning and muscle mass loss in early adolescents, though further controlled trials are necessary to confirm its efficacy.

**Abstract:**

**Background/Objectives**: This study evaluated the impact of short-duration physical exercise sessions and daily exercise recordings on the physical health and psychological health of junior high school students in Japan. **Methods**: A 3-month longitudinal school-based intervention initially enrolled 235 students, and the final analysis included 127 students who completed the program. The study was conducted using a single-arm pre–post design without a control group. The intervention included biweekly group exercise sessions and daily activity recordings. Students were separated into two groups based on their screen time (≥4 h per day or <4 h per day). **Results**: After the intervention, a significant decrease in screen time was observed in the group with baseline screen time of 4 h or more, whereas the group with baseline screen time of less than 4 h showed an increase in daily screen time. The results indicated a significant increase in body mass index and trunk muscle mass in both groups. For psychological measures, including the General Self-Efficacy Scale and Communicative and Critical Health Literacy scale, no significant changes over time or group × time interactions were observed, although post-intervention scores were significantly higher in the lower screen time group compared to the higher screen time group. However, significant changes in skeletal muscle index, eHealth literacy, and sleep duration were not observed. **Conclusions**: Due to the lack of a control group, these findings remain preliminary, and observed changes cannot be definitively attributed solely to the intervention. However, the program showed potential in reducing excessive screen time and increasing trunk muscle mass. Future randomized controlled trials are required to confirm the true efficacy of the intervention.

## 1. Introduction

Inactivity among young individuals has become a global issue. According to a World Health Organization survey of 1.6 million individuals between 11 and 17 years of age, approximately 81% indicated that they did not participate in moderate-intensity to high-intensity physical activity for at least 1 h per day [1]. Similarly, a survey of 980,000 junior high school students in Japan indicated declining physical activity [2]. The main causes of this decline included decreased time spent exercising and increased screen time (watching television, using smartphones, and playing video games) [2]. Additionally, this decline has resulted in concerns about poor health during adulthood. Physical inactivity not only increases the risk of obesity and lifestyle-related diseases [3] but also may negatively affect academic performance and mental health [4,5].

Screen time refers to the amount of time an individual spends using electronic or digital media devices such as televisions, smartphones, tablets, and computers [6]. Excessive screen time can lead to obesity, depression, sleep disorders, and other health problems in children and adolescents [7,8]. The Survey on Internet Usage Environment of Young People conducted by the Children and Families Agency in Japan reported that the average daily Internet usage times during weekdays were 3 h and 46 min for elementary school students, 4 h and 42 min for junior high school students, and 6 h and 14 min for high school students [9]. However, these times excluded television viewing; therefore, they could be higher.

Lifestyle disruptions, such as lack of exercise and long screen time periods, can have a negative impact on academics and self-efficacy [10,11]. Therefore, the health effects of inactivity and long screen time periods should not be ignored. To counteract this trend, a wide variety of exercise modalities have been examined, and network meta-analyses suggest that combining standard routines with diverse programs can effectively promote physical fitness in adolescents [12]. Thus, diverse physical education programs should be implemented in schools and exercise opportunities should be provided in the community so that children can develop good exercise habits that continue into adulthood [1].

Incorporating short periods of exercise into a routine is considered an effective means of establishing exercise habits and maintaining or improving health [13]. For instance, structured classroom-based short physical activity programs have demonstrated high feasibility and acceptance without disrupting school schedules [14]. Moreover, high-intensity anaerobic interval training can yield significant cardiorespiratory and muscular benefits in a substantially shorter duration than traditional moderate exercise, making it a highly time-efficient approach for school curricula [15]. Additionally, recording exercise details can enhance self-management skills and increase the motivation to exercise [16]. In fact, while the increased workload on teachers poses a barrier to traditional school-based intervention programs, using digital tools to track physical activity is recognized as a key factor in boosting students’ motivation and adherence [17].

Schools are the most efficient and equitable setting for providing uniform opportunities for physical activity to all students, regardless of their family background or individual motivation, and for promoting changes in health behaviors. However, the impact of performing short periods of exercise and recording daily exercise as part of a school-based physical education program on the psychological health and physical health of junior high school students has not been fully investigated. Clarification of the results of an intervention comprising short-duration exercise and recording exercise data may contribute to the development of instructional methods and educational programs that promote the health of junior high school students. Therefore, this study aimed to verify the effectiveness of such an intervention that combines short-duration physical exercise sessions and daily exercise recordings for junior high school students.

## 2. Materials and Methods

### 2.1. Study Design and Participants

This 3-month longitudinal study was conducted between November 2024 and January 2025. It included 235 students from a municipal junior high school in Fukushima City, Fukushima Prefecture, Japan. At the end of the study period, 127 students were included in the analysis because 11 students who refused to provide information, 27 students who did not submit consent forms, and 70 students with incomplete questionnaire data were excluded. The reason why 70 respondents did not return the survey was that the survey was not administered in two classes due to the busy end-of-semester period; this was not due to any bias toward a specific group or motivation level.

Screen time was defined as the total time spent using cell phones, watching television, and playing video games. At the beginning of the study, 51 participants comprised the group with ≥4 h of screen time and 76 comprised the group with <4 h of screen time. These screen time periods were chosen based on previous studies that have reported the importance of the 4-h separation period to the risk of developmental delay among infants [18], obesity risk among elementary and junior high school students [19], and stress among elementary and junior high school students [20]. Specifically, the participants were asked to perform short exercise sessions and record their daily exercise for 3 months. The associated changes in psychological factors and physical health indicators were investigated. This study was approved by the Ethical Review Committee of Fukushima Medical University on 25 December 2024 (approval number: REC 2024-150). Prior to the study, the participants and their parents or guardians were provided with a detailed written explanation. Written informed consent was obtained from the parents or guardians, and written informed assent was obtained from the participants (minors) themselves.

### 2.2. Exercise and Exercise Records

This health support project was launched with the cooperation of Junior High School A in Fukushima City to establish healthy lifestyle habits and improve self-efficacy. During this project, short periods of exercise and daily recordings of that exercise were performed during school hours for 3 months.

Each short exercise session was performed for 15 to 20 min once every 1 to 2 weeks. A total of 14 sessions were conducted during the 3-month period. Exercise was led by a researcher who is a licensed physical therapist with expertise in pediatric physical therapy and sports science. Exercise was performed in the school gymnasium after instructions were provided by a researcher involved in this study. All participants performed the exercise simultaneously. Since the exercise was conducted during regular school hours with all participants taking part simultaneously, attendance was extremely high, excluding those absent due to illness. However, individual attendance records for each of the 14 sessions were not strictly monitored or aggregated as quantitative research data. Three to six staff members (co-researchers and volunteer staff) participated in each exercise session by walking around the gymnasium and providing encouragement to all participants as they exercised. The participants performed aerobic exercises such as foot stomping and jumping, resistance training, and stretching. The exercise intensity target was a heart rate of 120 to 130 beats per minute, which is considered moderate intensity, with slight sweating [21]. During each exercise session, health-related topics such as the importance of sleep, diet, exercise, and lifestyle modifications were discussed.

Daily exercise records were maintained for 3 months using paper as well as an iPad (7th generation, MW742J/A, OS version 17.5.1; Apple, Cupertino, CA, USA) to allow reflection and consideration of lifestyle habits over time. The iPads were provided by the Board of Education. Data regarding upper-body and lower-body exercises, full-body exercises, club activities, and lessons were recorded. Whether the exercises were performed during each session was recorded; however, the duration and intensity of the exercise were not recorded. The exercise records could be viewed at any time so that the students could reflect on their daily activities. To support this, school teachers encouraged the students to perform daily recordings during the morning and evening school hours, and they were allowed to make recordings at a later date if they forgot. However, despite these promotional efforts, the exact compliance and adherence rate (i.e., the percentage of completed daily records out of the total days) were not quantitatively monitored or calculated.

### 2.3. Measurements

Measurements of lifestyle habits, psychological health, and physical health were recorded before and after the exercise intervention. Lifestyle habits included sleep duration, daily screen time (watching television, using cell phones, and playing video games), and screen time after 10:00 p.m. Psychological assessments included the General Self-Efficacy Scale (GSES), Communicative and Critical Health Literacy (CCHL) scale, and eHealth Literacy Scale (eHEALS). Physical measurements included body mass index (BMI), skeletal muscle mass index (SMI), and trunk muscle mass.

#### 2.3.1. Self-Efficacy

Self-efficacy refers to an individual’s belief that they can adequately cope with difficult situations [22]. The GSES, which is a reliable assessment tool that is available in 33 languages and used worldwide, measures general trends of self-efficacy [23]. This scale was designed for the general population older than 12 years of age and consists of the following 10 items: (1) I can always manage to solve difficult problems if I try hard enough; (2) if someone opposes me, I can find the means and ways to get what I want; (3) it is easy for me to stick to my aims and accomplish my goals; (4) I am confident that I could efficiently deal with unexpected events; (5) thanks to my resourcefulness, I know how to handle unforeseen situations; (6) I can solve most problems if I invest the necessary effort; (7) I can remain calm when facing difficulties because I can rely on my coping abilities; (8) when I am confronted with a problem, I can usually find several solutions; (9) if I am in trouble, I can usually think of a solution; and (10) I can usually handle whatever comes my way. Each of these items is rated using a 4-point Likert scale (1 = not at all true; 2 = somewhat true; 3 = moderately true; and 4 = exactly true). After summing up the scores of these 10 items, the total score ranged from 10 to 40; higher scores indicated better self-efficacy. Testing of the validity and reliability of the Japanese version of the GSES resulted in Cronbach’s alpha coefficient of 0.85, thus confirming its high internal consistency [24].

#### 2.3.2. Communicative and Critical Health Literacy

The CCHL scale is a three-level health literacy model consisting of functional health literacy (basic reading and writing skills), communicative health literacy (ability to apply new information), and critical health literacy (ability to critically analyze information regarding life events and situations) [25]. During the present study, health literacy was measured using the CCHL scale [26], which is a self-administered questionnaire for high school students and adults. This scale measures health literacy across functional levels by focusing on the ability to access, understand, and use health information. When using the CCHL scale, participants are asked whether they can perform the following: obtain health-related information from various sources; extract the required information; understand and communicate the information obtained; assess the reliability of the information; and make decisions based on the information obtained, specifically in the context of health-related issues. Each item is rated using a 5-point Likert scale (1 = strongly disagree; 2 = disagree a little; 3 = neither disagree nor agree; 4 = agree a little; and 5 = strongly agree). After summing up the scores of the five items, the total scores ranged from 5 to 25; higher scores indicated better communicative and critical health literacy. Cronbach’s alpha coefficient of the CCHL was 0.88, thus confirming its high internal consistency [26].

We selected the CCHL for this study because junior high school students, particularly those in their final years, increasingly encounter diverse health information in their daily and school lives. Currently, there is no validated Japanese health literacy scale specifically designed for junior high school students. Therefore, evaluating their communicative and critical health literacy—beyond basic functional literacy—using this established scale is crucial for understanding their health-making decisions as they transition into older adolescence.

#### 2.3.3. eHealth Literacy Scale

eHealth literacy refers to the ability to search for, evaluate, and use health information available through electronic media to make decisions [27]. eHEALS is a self-administered questionnaire for high school students and adults that consists of eight items that assess self-efficacy regarding the ability to search for, evaluate, and use health information. When using eHEALS, participants are asked whether they know how to access health information sites on the Internet, where to find useful health information websites, how to find useful health information websites on the Internet, how to use the Internet to answer questions about their health, how to use health information found online, how to evaluate health information sites on the Internet, how to distinguish between high-quality and low-quality health information websites on the Internet, and how to use information from the Internet to make decisions about their health status. Each item was rated using a 5-point Likert scale (1 = strongly disagree; 2 = disagree; 3 = neither disagree nor agree; 4 = agree; and 5 = strongly agree). After the scores of the five items were summed up, the total scores ranged from 8 to 40; higher scores indicated higher eHealth literacy. Cronbach’s alpha coefficient of the Japanese version of eHEALS was 0.93, thus confirming high internal consistency [28].

Due to the absence of eHealth literacy scales validated for Japanese junior high school students, the eHEALS was employed as the most appropriate tool. Assessing these digital-native students under the GIGA School Initiative using this scale is highly relevant to evaluate their ability to search for and evaluate online health resources.

#### 2.3.4. Physical Measurements

BMI, SMI, and trunk muscle mass were assessed using a multifrequency bioelectrical impedance analysis device (TANITA Corp., Tokyo, Japan). Measurements were conducted while the participants were in a standing position at rest. BMI was calculated as mass (kg) divided by height squared (m^2^). SMI was calculated as appendicular muscle mass (kg) divided by height squared (m^2^). Trunk muscle mass (kg) was recorded simultaneously using the bioelectrical impedance analysis device.

### 2.4. Statistical Analysis

Normality of all variables was confirmed using a histogram. The attributes of the participants were compared using the unpaired *t*-test and Χ^2^ test. The results were examined using a repeated-measures two-way analysis of variance and multiple comparison tests using the Bonferroni method. Partial eta-squared ($\eta_{p}^{2}$) was calculated as an effect size measure for the analysis of variance. Statistical analyses were performed using IBM SPSS Statistics version 30 with a significance level of 5%.

BMI, SMI, and trunk muscle mass were automatically excluded (listwise deletion) when values were missing (two participants in the group with ≥4 h of screen time and five participants in the group with <4 h of screen time were excluded) on the measurement days before and after the intervention. Therefore, the number of valid data points for each analysis did not match (Table 1, Table 2 and Table 3).

## 3. Results

Table 1 presents the participant characteristics, and Table 2 shows the analysis of variance for each parameter. Main effects and interactions between groups were observed for screen time, with a large effect size for both the main effect of group ($\eta_{p}^{2}$ = 0.344) and the interaction ($\eta_{p}^{2}$ = 0.120). Main effects between groups were observed for screen time after 10:00 p.m. ($\eta_{p}^{2}=0.121$, medium-to-large effect), GSES scores ($\eta_{p}^{2}=0.040$), and CCHL scale scores ($\eta_{p}^{2}=0.033$). Main effects before and after the intervention were observed for BMI and trunk muscle mass, showing a large effect for BMI ($\eta_{p}^{2}=0.557$) and a medium-to-large effect for trunk muscle mass ($\eta_{p}^{2}=0.088$). However, main effects between groups and before and after the intervention were not observed for sleep duration, eHEALS scores, and SMI, and no interactions were identified (all $\eta_{p}^{2}\leq 0.014$, indicating negligible or small effects).

Table 3 presents a comparison of parameters before and after the intervention. Multiple comparison tests showed that screen times decreased from an average of 368 min to 326 min for the ≥4 h group before and after the intervention and increased from 151 min to 190 min for the <4 h group. The GSES and CCHL scale scores did not differ between groups before the intervention, but they were significantly higher in the <4 h group after the intervention. SMI did not change in either group before or after the intervention, but BMI and trunk muscle mass increased significantly in both groups.

## 4. Discussion

### 4.1. Lifestyle

A systematic review of screen time for children 6 to 14 years of age categorized screen use into learning, entertainment (including watching videos and playing games), and social interaction and found that the main purpose of screen time for these children was entertainment rather than learning [29]. In the present study, the results of a 3-month intervention comprising short exercise sessions and recording daily exercise data to support health showed that the group with ≥4 h of screen time averaged 326 min of screen time after the intervention, which was 42 min less than that before the intervention, and that the group with <4 h of screen time averaged 190 min after the intervention, which was 39 min more than that before the intervention. According to the Survey on Internet Usage Environment of Young People conducted by the Children and Families Agency in Japan, the average screen time, excluding television viewing, per weekday was 4 h and 42 min for junior high school students [9]. In the group with ≥4 h of screen time, although we did not collect data on the specific types of electronic device use, our intervention may have contributed to a reduction in recreational screen time. However, this interpretation remains speculative. The 39-min increase in the group with <4 h of screen time may be attributed not only to the use of iPads as recorded during the main intervention at school but also to the cold winter weather—a characteristic of the intervention period—which likely led to a decrease in outdoor activities and an increase in indoor activities, resulting in a natural rise in activities such as watching TV and using smartphones.

Among children 15 to 17 years of age in the United States between 2021 and 2023, 55.0% had ≥4 h of screen time per day on weekdays for noneducational purposes; however, among children 12 to 14 years of age, 45.6% had ≥4 h of screen time per day on weekdays for noneducational purposes [30]. In another study, children with ≥4 h of screen time were more likely to experience anxiety (27.1% vs. 12.3%) and depressive symptoms (25.9% vs. 9.5%) within 2 weeks compared to those with <4 h of screen time [30]. Excessive screen time can lead to health problems such as depression and sleep disturbances [7,8]. In this study, 40.2% (51 participants) of the children had ≥4 h of screen time, which was similar to the rates reported by previous studies. Therefore, ongoing follow-up is warranted, especially for individuals with excessive screen time, to assess anxiety and depressive symptoms.

Sleep duration did not differ between the two groups, and screen time after 10:00 p.m. was longer in the group with ≥4 h of screen time, indicating that more of these children watched screens until bedtime compared to those in the group with <4 h of screen time. Screen time for children before bedtime negatively affects sleep quality and duration [31] and is associated with daytime sleepiness and irritability [32]. In this study, screen time after 10:00 p.m. after the intervention did not differ from that before the intervention in both groups. However, when providing health support to junior high school students, improving lifestyle habits, such as sleep and diet, is important.

### 4.2. Self-Efficacy and Health Literacy

Self-efficacy and health literacy are distinct concepts, but both are closely related to healthy living [33,34]. Health literacy refers to the ability to identify, understand, and use correct information about health, whereas self-efficacy refers to the confidence or belief that one can take action based on that information. Increased health literacy can increase self-efficacy and promote healthy behaviors [34]. Therefore, health education is important to increasing health literacy and fostering self-efficacy of students and allows them to choose and perform behaviors that promote their own health [35].

The results of the between-group comparison after the intervention showed differences in GSES and CCHL scale scores of the two groups. The differences in self-efficacy and health literacy between the groups with ≥4 h or screen time and <4 h of screen time after the intervention were clear and indicated that brief exercise sessions and recording exercise data may have positive psychological effects. GSES and CCHL scale scores had no main effects before or after the intervention; however, a closer look at the results showed that after the intervention, the reduction in screen time in the group with ≥4 h of screen time suggested a change in daily activities during the day. However, the specific details are unknown. Junior high school students experience physical and psychological changes induced by puberty that can be perplexing. Although they often become more concerned and anxious about their bodies, many have not yet developed the ability to think objectively about their health [36]. The fact that the intervention resulted in slight changes suggested that it may be necessary to consider the possibility of both positive and negative changes and that health education should be structured accordingly.

### 4.3. Muscle Mass

A study of school-based exercise for elementary school students showed improved lean tissue mass and muscle strength compared to those of a control group [37,38]. Additionally, 2 months of high-intensity training during physical education classes for junior high school and high school students improved grip strength and SMI compared to those of a control group in another study [39]. Thus, implementing daily exercise programs and physical education classes in schools may improve the muscle mass and strength of children. In the present study, BMI and trunk muscle mass of both groups increased after 3 months, whereas SMI did not change. The duration of exercise during this study was shorter than that of previous studies. Our intervention included a total of 14 exercise sessions that each lasted 15 to 20 min. Because there was no control group, it is unclear whether the increase in BMI and trunk muscle mass was caused by physical growth over the 3-month period, seasonal variations, or the effects of exercise on the participants. The intervention period was during the winter months of November through January, a season when children’s physical activity levels generally decline, making them prone to weight and BMI gain and muscle mass loss. Given these circumstances, while it is difficult to separate the effects of the intervention from natural growth and development and seasonal variations, the observation that participants were able to maintain and improve their trunk muscle mass without reducing SMI suggests that regular exercise interventions—even if brief—could potentially serve as a buffering effect against the decline in physical activity during the winter months. Nevertheless, these conclusions must be interpreted with caution due to the lack of a control group.

### 4.4. Limitations

This study is a valuable report that utilized the infrastructure (iPads) of Japan’s “GIGA (Global and Innovation Gateway for All) School Initiative” to clarify the relationship between screen time and changes in children’s health behaviors. However, it has some limitations. First, as it was conducted in collaboration with a junior high school, this study had to exclude numerous participants who failed to complete the questionnaires in two of the classes owing to the hectic end-of-semester period. Specifically, the final analysis included only 127 of the 235 initially enrolled students (54% of the original sample). This substantial loss of participants (46% attrition rate) may affect the representativeness of the findings and limit the generalizability of our results. Second, although the CCHL and eHEALS were originally developed for high school students and older individuals, and their validity for junior high school students remains unverified, they nonetheless provide valuable insights. Currently, Japan lacks validated health literacy scales specifically for this younger cohort. Given that modern students are digital natives under the GIGA School Initiative, evaluating these students’ critical and digital health literacy is essential, marking a meaningful first step in assessing this population. Third, although this was a longitudinal study, the interpretation of changes in BMI and trunk muscle mass in both groups was limited because there was no control group. Furthermore, it is possible that the 5 to 10 min spent each day on the iPad for journaling and reflection were included in the measured “daily screen time.” Further interventions should be adapted for use in Japanese schools and control groups should be included for comparisons.

## 5. Conclusions

This study examined the impact of short periods of exercise and daily exercise recordings on lifestyle habits, psychological health, and physical health of junior high school students. Due to the methodological limitation of a single-arm pre–post design without a control group, the findings of this three-month program remain preliminary. While a reduction in screen time in the group with ≥4 h of screen time and an increase in trunk muscle mass in both groups were observed, it is not possible to definitively attribute these changes solely to the intervention rather than to natural growth or seasonal factors. Furthermore, no broad impact on general self-efficacy or health literacy was observed, suggesting that a more targeted psychological approach is needed for mental health transformation. Future randomized controlled trials with an appropriate control group are required to confirm the true efficacy of this program.

## Figures and Tables

**Table 1:** Participant characteristics.

	All (*n* = 127)	Group with ≥4 h of Screen Time (*n* = 51)	Group with <4 h of Screen Time (*n* = 76)	*p* Value
Age, year	13.4 (1.1)	13.3 (1.1)	13.5 (1.0)	0.855
Sex (male, female), *n*	61, 66	25, 26	36, 40	0.148
Height, cm	158.2 (7.4)	158.4 (6.3)	158.2 (8.1)	0.869
Weight, kg	50.4 (10.8)	50.0 (11.1)	50.6 (10.7)	0.729

Data are presented as the mean (standard deviation). *p* < 0.05 was considered significant.

**Table 2:** Analysis of variance of each parameter.

Parameter	Source of Variation	*F*	df	*p*	$\eta_{p}^{2}$
Sleep duration	Before and after intervention	0.683	1	0.410	0.005
	Groups	0.022	1	0.882	<0.001
	Interaction	1.715	1	0.193	0.014
Daily screen time	Before and after intervention	0.033	1	0.856	<0.001
	Groups	65.540	1	<0.001	0.344
	Interaction	17.113	1	<0.001	0.120
Screen time after 10:00 p.m.	Before and after intervention	0.128	1	0.721	0.001
	Groups	17.151	1	<0.001	0.121
	Interaction	1.336	1	0.250	0.011
GSES	Before and after intervention	1.821	1	0.180	0.014
	Groups	5.163	1	0.025	0.040
	Interaction	0.234	1	0.629	0.002
CCHL	Before and after intervention	0.547	1	0.461	0.004
	Groups	4.271	1	0.041	0.033
	Interaction	0.738	1	0.392	0.006
eHEALS	Before and after intervention	1.004	1	0.318	0.008
	Groups	0.004	1	0.950	<0.001
	Interaction	0.094	1	0.760	0.001
BMI	Before and after intervention	148.459	1	<0.001	0.557
	Groups	0.149	1	0.700	0.001
	Interaction	0.107	1	0.745	0.001
SMI	Before and after intervention	0.044	1	0.833	<0.001
	Groups	0.753	1	0.387	0.006
	Interaction	0.163	1	0.688	0.001
Trunk muscle mass	Before and after intervention	11.350	1	0.001	0.088
	Groups	0.013	1	0.911	<0.001
	Interaction	0.044	1	0.834	<0.001

*p* < 0.05 was considered significant. The number of participants included in the analysis of sleep duration, screen time, screen time after 10:00 p.m., GSES scores, CCHL scale scores, and eHEALS scores was 127 (51 in the group with ≥4 h of screen time and 76 in the group with <4 h of screen time). The analysis of BMI, SMI, and trunk muscle mass included 49 participants in the group with ≥4 h of screen time and 71 participants in the group with <4 h of screen time. BMI, body mass index; CCHL, Communicative and Critical Health Literacy; eHEALS, eHealth Literacy Scale; GSES, General Self-Efficacy Scale; SMI, skeletal muscle mass index.

**Table 3:** Comparison of parameters before and after intervention.

	Before Intervention		After Intervention	
	Group with ≥4 h of Screen Time	Group with <4 h of Screen Time	Group with ≥4 h of Screen Time	Group with <4 h of Screen Time
Sleep duration, minutes	452 (71)	456 (53)	461 (59)	454 (50)
Daily screen time, minutes	368 (178)	151 (54) *	326 (181) ^†^	190 (109) *^,†^
Screen time after 10:00 p.m., minutes	81 (105)	27 (37) *	73 (97)	32 (40) *
GSES score, points	24.7 (5.1)	26.5 (5.2)	25.1 (5.6)	27.3 (6.0) *
CCHL scale score, points	17.9 (2.7)	18.8 (3.5)	17.4 (4.2)	18.8 (3.8) *
eHEALS score, points	24.9 (5.9)	24.6 (7.3)	25.3 (7.0)	25.4 (7.6)
BMI, kg/m^2^	19.8 (3.6)	20.1 (3.6)	20.5 (3.5) ^†^	20.7 (3.4) ^†^
SMI, kg/m^2^	6.7 (0.9)	6.9 (0.8)	6.7 (1.0)	6.8 (0.9)
Trunk muscle mass, kg	19.5 (2.7)	19.5 (2.6)	19.8 (2.6) ^†^	19.7 (2.6) ^†^

Data are presented as the mean (standard deviation). * Group with ≥4 h of screen time vs. group with <4 h of screen time. ^†^ Before intervention vs. after intervention. *p* < 0.05 was considered significant. The number of participants included in the analysis of sleep duration, screen time, screen time after 10:00 p.m., GSES scores, CCHL scale scores, and eHEALS scores was 127 (51 in the group with ≥4 h of screen time and 76 in the group with <4 h of screen time). The analysis of BMI, SMI, and trunk muscle mass included 49 participants in the group with ≥4 h of screen time and 71 participants in the group with <4 h of screen time. BMI, body mass index; CCHL, Communicative and Critical Health Literacy; eHEALS, eHealth Literacy Scale; GSES, General Self-Efficacy Scale; SMI, skeletal muscle mass index.

## Data Availability

The data presented in this study are available on Figshare at https://doi.org/10.6084/m9.figshare.29203586.

## Abbreviations

The following abbreviations are used in this manuscript:

GSES	General self-efficacy scale
CCHL	Communicative and critical health literacy
eHEALS	eHealth literacy scale
BMI	body mass index
SMI	Skeletal muscle mass index
